# Axon initial segment potassium channel density in cortical neurons

**DOI:** 10.1186/1471-2202-16-S1-P295

**Published:** 2015-12-18

**Authors:** Wen Zhang, Boqiang Fan, Ping Zheng, Yuguo Yu

**Affiliations:** 1 The State Key Laboratory of Medical Neurobiology and Institutes of Brain Science, School of Life Sciences, Fudan University, Shanghai, 200433;China

## 

There is a growing interest in estimating actual density ranges of Na+ channels in the very thin axon, especially in the action potential (AP) initiation zone, i.e., the axon initial segment (AIS, 20-50 microns away from the cell body). Both immunostaining studies and patch-clamp recordings indicated a relatively high density of Na+ channels in AIS of either pyramidal regular-spiking (RS) cells [1] or fast-spiking (FS) GABAergic interneurons [2,3]. Here, we investigated potassium channel densities in AISs of both RS and FS cells in same recording conditions.

**Figure 1:**
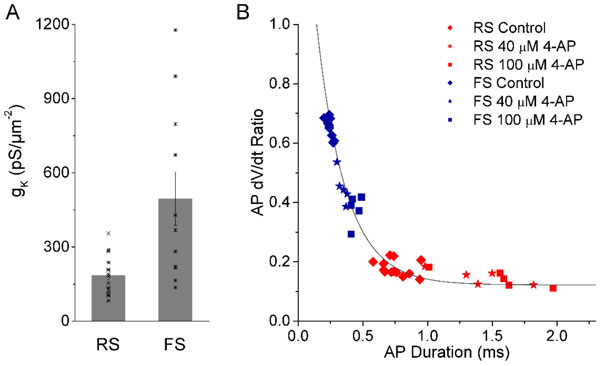
**A. The bar graph shows peak K+ conductance density recorded in axon initial segment of cells (16 recording axons for RS pyramidal cell, 11 for FS interneuron) by outside-out axon patch recording technique**. B. Summary of the results from partially blocking K+ channels. The AP dV/dt ratio is an exponentially decaying function of the spike duration.

Our axonal recordings directly revealed that there is a very lower potassium density gK = 185.8±19 pS/µm2 N = 16) for the RS AIS while a higher gK (495.7±108 pS/µm2, N = 11) for FS AIS, see Figure 1A. For both the RS pyramidal cells and FS PV cells, partially blocking K+ channels by applying 4-AP broadened the spike duration and decreased the dV/dt ratio significantly (P < 0.05) (For RS cells: N = 5; For RS cells: N = 4). Interestingly, we observed that the AP dV/dt ratio is an exponentially decaying function of the spike duration for both RS- and FS-spikings (see Figure 1B), such that y = 0.12+0.16EXP((0.5-x)/0.2), where y represents the dV/dt ratio and × represents the AP duration. These observations suggest strongly that potassium channel density is one of the major intrinsic factors dominating the spike shape properties, especially half-height spike duration and dV/dt ratio.

In sum, the significant difference in potassium channel density in axonal initial segment where action potentials are initiated may play a critical role in controlling action potential properties of both RS- and FS-spiking cells in nervous system by the same general biophysical rule. These results may be important for constructing computational models of different types of cortical neurons.
